# Identification and Expression Analysis of R2R3-MYB Transcription Factors Associated with Flavonoid Biosynthesis in *Panax quinquefolius*

**DOI:** 10.3390/ijms25073709

**Published:** 2024-03-26

**Authors:** Guimei Song, Yan Yan, Chun Guo, Jiankang Chen, Yumeng Wang, Yingping Wang, Jiaxin Zhang, Chang Gao, Junmei Lian, Xiangmin Piao, Peng Di

**Affiliations:** State Local Joint Engineering Research Center of Ginseng Breeding and Application, Jilin Agricultural University, Changchun 130118, China; sgm9864@outlook.com (G.S.); yany@jlau.edu.cn (Y.Y.); gc2069662120@outlook.com (C.G.); chen_jk0606@outlook.com (J.C.); wym3027456855@outlook.com (Y.W.); wangyingping@jlau.edu.cn (Y.W.); zhangjiaxin999@outlook.com (J.Z.); gaochang9999@outlook.com (C.G.); lian@mails.jlau.edu.cn (J.L.)

**Keywords:** *Panax quinquefolius* L., R2R3-MYB, transcription factor, secondary metabolites

## Abstract

*Panax quinquefolius* L. is an important medicinal plant, and flavonoids are among its main secondary metabolites. The R2R3-MYB transcription factor plays an irreplaceable role in plant growth, development, and secondary metabolism. In our study, we identified 159 R2R3-MYBs and analyzed their physical and chemical properties in *P. quinquefolius*. The protein length of 159 *PqMYB*s varied from 107 to 1050 amino acids. The molecular weight ranged from 12.21 to 116.44 kDa. The isoelectric point was between 4.57 and 10.34. We constructed a phylogenetic tree of *P. quinquefolius* and *Arabidopsis thaliana* R2R3-MYB family members, and *PqMYB* members were divided into 33 subgroups. Transcriptome data analysis showed that the expression patterns of *PqMYB*s in root, leaf, and flower were significantly different. Following the MeJA treatment of seedlings, five candidate PqMYB genes demonstrated a response. A correlation analysis of *PqMYB*s and candidate flavonoid pathway genes showed that *PqMYB2*, *PqMYB46*, and *PqMYB72* had correlation coefficients that were higher than 0.8 with *PqCHS*, *PqANS4*, and *PqCCoAMT10*, respectively. Furthermore, a transient expression assay confirmed that the three *PqMYBs* were localized in the nucleus. We speculated that these three *PqMYB*s were related to flavonoid biosynthesis in *P. quinquefolius*. These results provided a theoretical basis and a new perspective for further understanding the R2R3-MYB gene family and the biosynthesis mechanism of secondary metabolites in *P. quinquefolius*.

## 1. Introduction

*Panax quinquefolius* is an herb that belongs to the *Panax* classification and which has been used for centuries due to its various pharmacological functions [1,2]. It is native to Montreal, Vancouver Mountain, and Quebec in Canada, and to the eastern United States. *P. quinquefolius* has been shown to have a protective effect on myocardial function, as well as the ability to lower blood sugar levels and blood pressure. Additionally, it has demonstrated promise in the field of cancer prevention and treatment. The extensive pharmacological benefits of *P. quinquefolius* make it a valuable subject for further research and exploration [3]. The chemical constituents in *P. quinquefolius* are complex, including saponins, flavonoids, volatile oils, amino acids, polysaccharides, and so on [4].

In plants, flavonoids are among the main secondary metabolites that have a number of biological activities, and they play a crucial role in the growth and development of plants [5]. To date, more than 9000 flavonoids have been detected in plants [6]. They can be divided into flavonoids, isoflavones, flavonols, flavanones, proanthocyanidins, and anthocyanins [7]. They have antioxidant, anti-inflammatory, antibacterial, and anticancer effects which are beneficial to human health [8]. Panasenoside was identified for the first time in *P. quinquefolius* by Zhang et al. [9]. Meng et al. found that the content of total flavonoids in different tissues of *P. quinquefolius* was variable; the highest content of flavonoids was found in flowers, and the lowest was found in roots [10]. MYB transcription factors are abundant in plants [11]. The MYB domain is mainly composed of about 51 amino acids as a repetition, and they the highly conserved two peptide segments to form three alpha helices. The second and third segments of the MYB domain create a three-dimensional helix–turn–helix (HTH) structure through three regularly spaced tryptophan residues, enabling them to bind to the major DNA groove at a specific recognition site during transcriptional processes [12]. Every MYB transcription factor consists of one to four repetitive MYB domains [13]. Based on the number of domain repeats, different transcription factors containing MYB are mainly divided into the following four types: 1R-MYB types (one or two separated repeats), the 2R-MYB type (R2R3-MYB, two adjacent repeats), the 3R-MYB type (three adjacent repeats), and the 4R-MYB type (consisting of four adjacent repeats) [14]. R2R3-MYB has the largest proportion, and plant growth, development, metabolic regulation and stress response cannot be separated from it [15,16]. The R2R3-MYB gene family carry out conservative regulatory function in terrestrial plants, particularly for the biosynthesis process of flavonoids [17].

With the completion of genome sequencing for many plants, more and more R2R3-MYB gene members have been identified in medicinal plants. For example, 95 were found in *Scutellaria baicalensis* [18], 87 were found in *Gynostemma pentaphyllum* [19], and 101 were found in *Apocynum Venetism* [20]. Understanding the function of the R2R3-MYB transcription factor will enrich our comprehension of the transcriptional regulation mechanism of plant secondary metabolism [21]. Plant R2R3-MYB has been gradually studied regarding the regulation of flavonoid biosynthesis. In *S. baicalensis*, *SbMYB45* and *SbMYB86.1* activated the response of *SbCHI* to light at the transcription level and increased the content of flavonoids in *S. baicalensis* [22]. *EsMYBA1* in *Epimedium sagittatum* can interact with bHLH regulators in various flavonoid pathways, and the promoters of DFR and ANS were activated by *EsMYBA1* [23]. *GpMYB81* was considered to be a dual-function transcription factor which can not only regulate the biosynthesis of gypenosides in *G. pentaphyllum*, but also regulate the content of flavanols [19]. The overexpression of *Chrysanthemum morifolium CmMYB3* in tobacco and *A. thaliana* can increase the content of flavonoids [24]. In *Fragaria* × *ananassa*, FaMYB5 was an R2R3-MYB activator involved in the composition of MBW, which positively regulates the biosynthesis of anthocyanin and proanthocyanidin [25]. In *Tetrastigma hemsleyanum,* the hairy roots overexpressing *ThMYB4* and *ThMYB7*, a significant enhancement in the transcriptional levels of *ThCHS* and *ThCHI* was observed, suggesting that *ThMYB4* and *ThMYB7* potentially function as regulatory components in flavonoid biosynthetic pathways [26]. However, research on the MYB transcriptional regulation of flavonoid biosynthesis in *P. quinquefolius* is still drawing a blank.

Plant hormones participate in various physiological processes and mediate responses to biotic and abiotic stresses [27]. MeJA is an important plant hormone which can mediate communication within and between plants and regulate plant defense responses [28]. MeJA can improve the biosynthesis of secondary metabolites by stimulating the corresponding transcription factors in plants. In ginseng, MeJA has been observed to induce the expression of *PgMYB2*, upregulating PgDDS and promoting the accumulation of ginsenosides [29]. It has been reported that MeJA could improve the accumulation of flavonoids in the callus of *Pyrus communis* [30]. In *Salvia miltiorrhiza*, *SmMYB2* is a MeJA-induced transcription factor which can positively regulate the formation of salvianolic acid [31]. In this study, MeJA was used to treat *P. quinquefolius* seedlings, and the transcriptome data under the condition of hormone treatment at the seedling stage were analyzed. It was found that most R2R3-MYB genes had different expression patterns in response to MeJA.

We identified 159 PqMYBs from *P. quinquefolius* in total in this study. To predict the PqMYB genes that regulate flavonoid compounds, a co-expression analysis was conducted on the flavonoid synthesis-related subfamily of PqMYB genes and the pathway genes associated with the flavonoid biosynthetic pathway. Additionally, the responses of PqMYB genes and those pathway genes highly correlated with the flavonoid pathway under MeJA treatment were also investigated. This comprehensive study serves as a solid foundation for further exploration into the functionality of the *PqMYB*s. Moreover, it offers valuable insights into the transcriptional regulation mechanism underlying flavonoid biosynthesis in *P. quinquefolius*.

## 2. Results

### 2.1. Identification and Physicochemical Properties Analysis

In this study, 354 *PqMYBs* which had complete MYB domains were identified on the basis of the *P. quinquefolius* genome. Then, 159 *PqMYB*s containing two R2R3 repeat domains were screened using Pfam and SMART (Supplementary Table S1). Overall, 159 *PqMYB*s were renamed as *PqMYB1*-*PqMYB159*; we carried out an in-depth analysis of the information of the 159 *PqMYB* genes, including the length of protein, molecular weight (MW), and isoelectric point (pI). The protein length of *PqMYB* ranged from 107 (*PqMYB5*) to 1050 (*PqMYB50*)aa, MW from 12.21 (*PqMYB5*) to 116.44 kDa (*PqMYB125*), and PI from 4.57 (*PqMYB115*) to 10.34 (*PqMYB77*) (Supplementary Table S2).

### 2.2. Phylogenetic Tree, Chromosome Location, and Collinear Analysis

**Figure 1:**
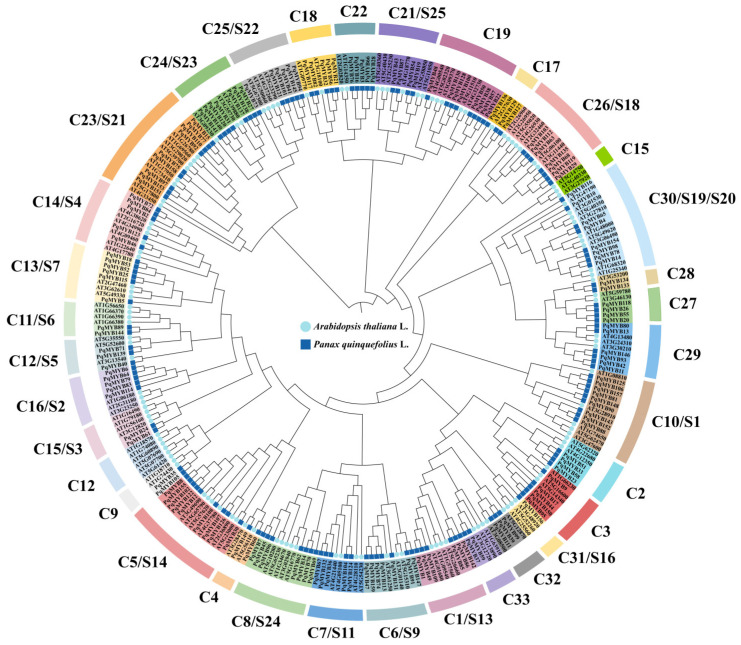
Phylogenetic analysis of R2R3-MYB proteins in *P. quinquefolius* and *A. thaliana*. The phylogenetic tree was created using IQ-tree with 1000 bootstrap replications. The phylogenetic tree is divided into 36 subfamilies, labelled with protein gene names in different background colors, with the corresponding subfamily names marked in the outer circle. The inner circles are indicated by light blue circles for *A. thaliana* and blue squares for *P. quinquefolius* MYB proteins, respectively.

According to the conservation of amino acid sequences of 126 *AtR2R3-MYB* proteins in *A. thaliana*, we constructed a phylogenetic tree with 159 amino acid sequences of *PqMYB* proteins (Figure 1), which we divided into 33 subgroups according to sequence similarity and topological structure. The 159 *PqMYB* transcription factors were clustered into 33 subgroups (named C1–C33), among which no genes clustered with *A. thaliana* were found in subgroups S10, S12, or S15. In addition, MYB members in *P. quinquefolius* were distributed in other subfamilies to varying degrees. The members of PqMYB were mainly distributed in C5(S14), C10(S1), C13(S7), C22, C24(S23), C25(S22), and C29(S10). In these subfamilies, the number of R2R3-MYB members of *P. quinquefolius* was about 1.5–2.5 times that of *A. thaliana*. From this, we can see that *PqMYBs* and *AtMYBs* have some common ancestors which have undergone specific amplification and differentiation after being separated in the evolutionary process. Thanks to the phylogenetic analysis of these two plants, we can understand the interspecific evolutionary relationship of the R2R3-MYB gene family and predict the function of unknown target genes.

**Figure 2:**
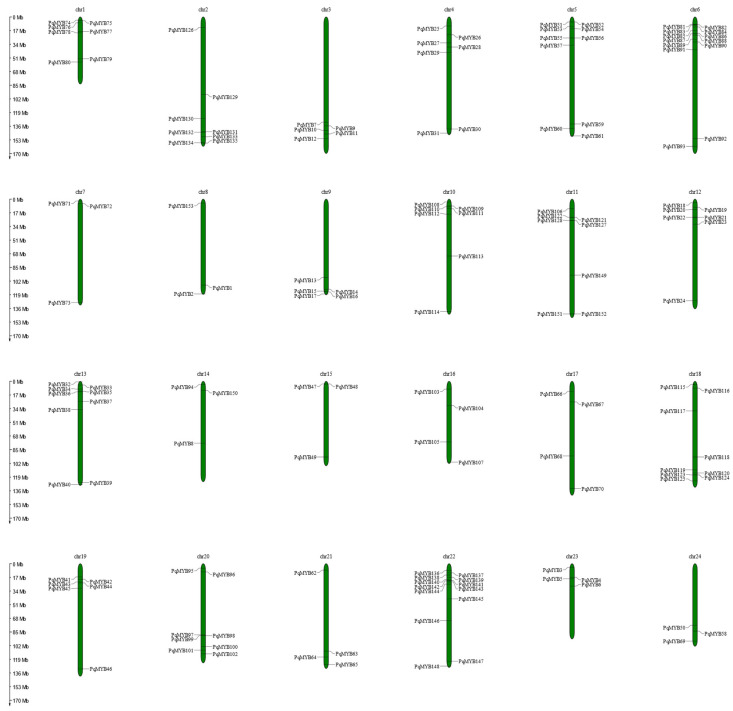
Chromosome location analysis of R2R3-MYB proteins in *P. quinquefolius*. There are 24 chromosomes (Chr1–Chr24) in *P. quinquefolius*. Gene positions and chromosome length were measured using the scale on the left in megabases (Mb).

The location of these *PqMYBs* was mapped to the chromosomes of *P. quinquefolius* using the online tool Mapgene 2 chrom (Figure 2). According to genome chromosome mapping analysis, among 159 *PqMYB*s, 153 MYB genes were distributed onto 24 chromosomes. Chromosomes 6 and 22 contained the most *PqMYB*s (13 members, accounting for 8.2%), followed by Chromosome 5, with 10 *PqMYB*s, while Chromosomes 7, 8, 14, and 24 contained the least number of genes: only three. The chromosome location analysis showed that the distribution of *PqMYB*s was uneven across chromosomes; most *PqMYB*s were found at the ends of chromosomes, and a few were located in the center, such as *PqMYB113*.

In this study, the duplicate *PqMYB*s were identified in the *P. quinquefolius* genome using BLSATP and MCScanX. We detected five pairs of tandem duplication events. Furthermore, results showed that 80% (126) of the *PqMYB*s were duplicated and retained from singleton duplication events, followed by 15% (24) from tandem duplication events, compared to only 1% (2) from singleton duplication events and proximal duplication events. These results suggested that some *PqMYB*s may be caused by gene singleton duplication events, and that tandem duplication also plays a key role. Different repetitive events may promote the expansion of the R2R3-MYB gene family in *P. quinquefolius* (Supplementary Table S3).

To further clarify the possible evolutionary relationship of *PqMYB*s between *P. quinquefolius* and two other representative species of *Panax*, *Panax ginseng* and *Panax notoginseng*, a collinear map of comparative relationships was constructed between *P. quinquefolius* and the other two representative species of *Panax* (Supplementary Figure S1A). We found that there were 137 and 41 orthologous gene pairs between *P. quinquefolius* and *P. ginseng* and *P. quinquefolius* and *P. notoginseng*, respectively. Compared with *P. notoginseng*, *P. ginseng* had more homologous gene pairs than *P. quinquefolius*, which indicates that the genome relationship between the two plants is closer. Of the three plants, these R2R3-MYB gene pairs may play a special evolutionary role.

KA/KS value can allow a better understanding of the evolutionary processes of plants and can be used to express the selection pressure and speed of species’ evolution. Generally, Ka/Ks < 1 indicates that the gene pair is undergoing a purifying selection, Ka/Ks > 1 indicates positive selection, and ka/Ks = 1 indicates neutral evolution. In this study, except for two pairs of homologous genes, *PqMYB64*-*PqMYB6* and *PqMYB126*-*PqMYB107*, Ka/Ks > 1, which indicated that the other 42 pairs of homologous genes had values less than one, which indicated that most *PqMYB*s have undergone purification selection pressure in *P. quinquefolius* (Supplementary Table S4).

### 2.3. Motifs and Domains of Protein and cis-Acting Element Analysis

**Figure 3:**
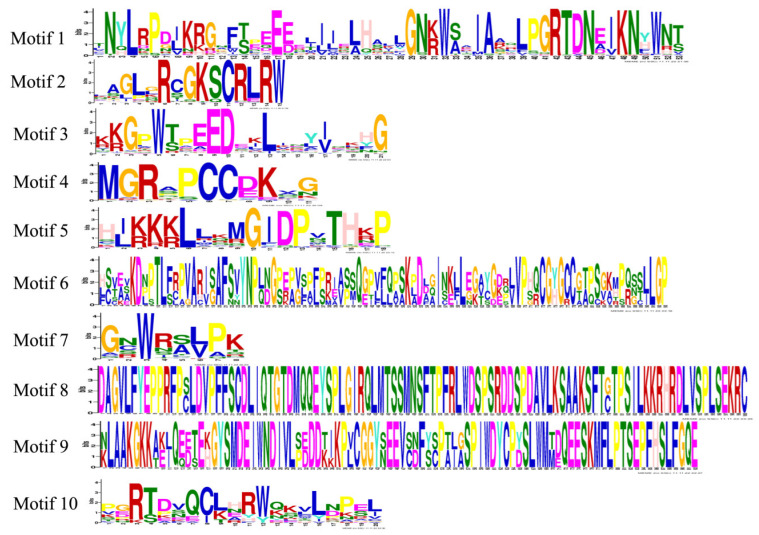
Logos of 10 motifs predicted for R2R3-MYB in *P. quinquefolius*. All 10 identified motifs are indicated by different colors and their respective sequence logos.

To further understand the diversity of the *PqMYB*s, we conserved the motif distribution of *PqMYB*s (Supplementary Figure S2A). Ten conserved motifs in *PqMYB* proteins were identified (Figure 3). At the N-terminal of the sequence, most protein sequences contained motifs -1, -2, and -3, indicating that motifs -1, -2, and -3 are primary components of the PqMYB protein sequence. In the evolution of this family, motifs -1, -2, and -3 have the most primitive structures and may have the most conservative functions.

The functions of gene family members are closely related to their structures, which can reflect the phylogenetic relationship within the gene family. Therefore, our study constructed a phylogenetic tree based on the 159 amino acid sequences of PqMYB to better understand their gene structures. The analysis of the gene structures showed that the number of introns in *PqMYB* ranged from 0 to 15. In total, 50.9% of the genes contained three introns. Some PqMYB had similar exon/intron numbers in the same group, such as *PqMYB50*, *PqMYB38*, *PqMYB22*, *PqMYB48*, and *PqMYB56* in the C19 subgroup. Some *PqMYB*s contained a large number of introns, such as *PqMYB53*, with 13 introns, *PqMYB38* with 14 introns, and *PqMYB55* with 12 introns (Supplementary Figure S2B).

**Figure 4:**
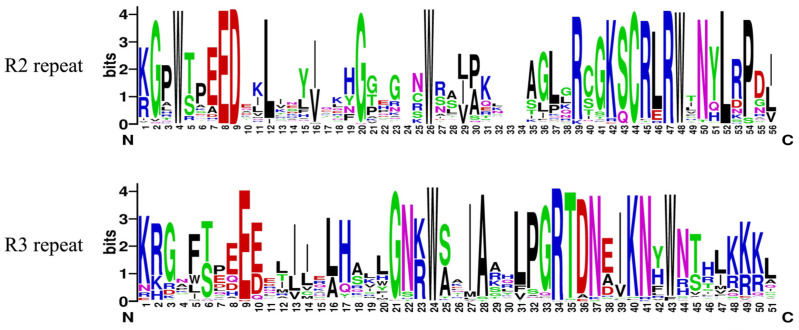
R2 and R3 repeats of the proteins of the R2R3-MYB family in *P. quinquefolius*.

To further acknowledge the characteristics of the conserved domain of *PqMYB*s and the R2 and R3 repeats, proteins were sequenced and analyzed. The sequence identification was generated by Weblogo (Figure 4). The results showed that the *PqMYB*s had the typical characteristics of conserved domains of MYB, and their R2 and R3 repeats contained 51–56 amino acid residues. R2 and R3 MYB repeats of *PqMYB*s contained characteristic amino acids, including three conserved and equidistant Trp (W) residues (W4, W26, W48) found in R2 repeats, which play an important role in MYB-DNA interaction by forming a hydrophobic core in the HTH structure (also known as the characteristics of the MYB domain). The first Trp residue (W5) in R3 repeats is usually replaced by Phe(F) or Leu(L), and only the second Trp (W24) and the third Trp (W43) are conserved.

A total of 47 cis-regulatory elements were found, most of which were related to hormone responses and abiotic/biotic stress. The light response element was the most common, which exists in the promoter region of 142 genes, followed by abscisic acid (ABA) (111), methyl jasmonate (MeJA) (109), Auxin (73), gibberellin (GA) (68) and salicylic acid (SA) (67) (Supplementary Figure S3). We also found that most of the cis-acting elements in *PqMYB* were involved in abiotic stress, including the TATA-box, CAAT-box, Box-4, MYB, MYC, G-box, ABRE (ABA response element), STRE (stress response element), and MBS. We also found that there are many oxidative defense elements such as ARE (antioxidant response element) (Supplementary Table S5). In addition, some cis-acting elements are associated with the biosynthesis of plant hormones and are mainly involved in the biosynthesis of MeJA (CGTCA motif and TGACG motif) and Auxin (TGA element). These results indicate that *PqMYB* is regulated by many factors.

### 2.4. Expression Pattern Analysis in Different Tissues

**Figure 5:**
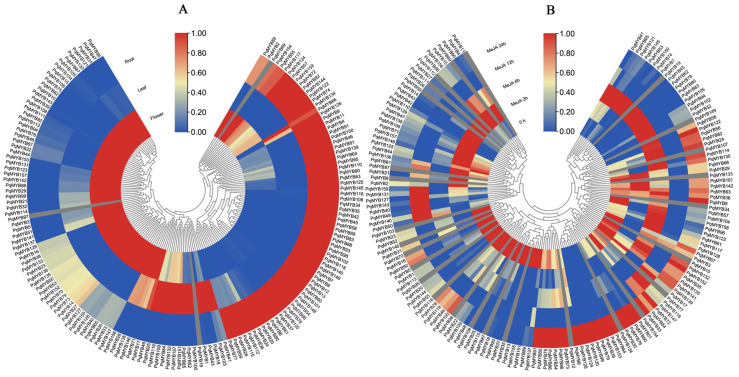
Expression patterns of root, leaf, and flower tissues and MeJA treatment for 0, 6, 12, and 24 h in *P. quinquefolius*. (**A**) Expression pattern of the R2R3-MYB gene in different tissues. (**B**) The expression pattern of the *PqMYB* gene treated with MeJA at a different time. The expression levels are illustrated using a blue–red scale. Blue indicates lower expression and red indicates higher expression.

The expression patterns of R2R3-MYB in three tissues (roots, leaves, and flowers) of four-year-old *P. quinquefolius* were analyzed (Supplementary Table S6) (Figure 5A). Red indicates upregulation and blue indicates downregulation. Among all *PqMYB*s, it was found that except for three genes (*PqMYB97*, *PqMYB69*, and *PqMYB19*), the transcripts of the other 156 *PqMYB*s were distributed in different tissues. In flowers, 76 *PqMYB*s were highly expressed. This was followed by roots, with a number of 66, and the number of genes highly expressed in leaves was the lowest, at only 17; these genes were specifically expressed. This suggests that different *PqMYB*s may be involved in regulating the different growth and development processes of *P. quinquefolius*. On the whole, the expression trends of the *PqMYB*s were tissue-specific, and we speculate that these expression patterns in *P. quinquefolius* will provide a new perspective for the further functional study of *PqMYB*s.

### 2.5. RNA-Seq and qRT-PCR

To investigate the response of PqMYB genes to MeJA treatment, we treated 8-week-old ginseng seedlings with MeJA and conducted transcriptome profiling. The results showed that 27 genes had no expression after MeJA treatment. Some *PqMYB* gene expression levels increased significantly, such as those of *PqMYB64*, *PqMYB79*, *PqMYB153*, *PqMYB126*, *PqMYB33*, and *PqMYB51*. Some other *PqMYB* gene expression levels also increased significantly, such as those of *PqMYB151*, *PqMYB48*, *PqMYB25*, *PqMYB155*, *PqMYB37*, etc. (Supplementary Table S7) (Figure 5B). Overall, the expression patterns of most genes showed significant changes, and a majority of genes exhibited varying degrees of response to the time-dependent changes induced by MeJA treatment.

**Figure 6:**
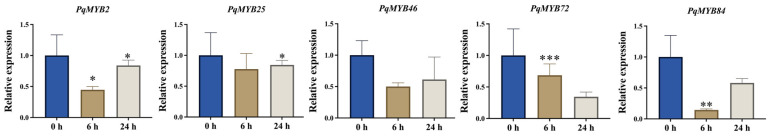
Relative expression of 5 R2R3-MYB transcription factors in leaves of *P. quinquefolius* treated with MeJA at different times for 0 h, 6 h, and 24 h. Data indicate the mean ± SD, and the dots represent raw data; *: *p* < 0.05, **: *p* < 0.01, ***: *p* < 0.001.

To further explore the response patterns of *PqMYB* that participate in the regulation of flavonoid biosynthesis in MeJA treatment, we selected five candidate *PqMYB*s at 0 h, 6 h, and 24 h for experimental validation with the transcriptome profiles. They all showed different expression patterns under MeJA treatment, which indicated that all five genes responded to MeJA induction (Figure 6). Among the five selected *PqMYB*s, four of them exhibited a downregulation-followed-by-upregulation pattern in response to MeJA treatment. Specifically, their relative expression levels decreased at 6 h and then increased at 24 h. However, only one MYB (*PqMYB72*) showed a continuous downregulation pattern at 6 h and 24 h. The qRT-PCR results of the five *PqMYB*s were consistent with the transcriptome analysis, indicating that candidate MYBs involved in regulating flavonoid biosynthesis displayed notable responses to MeJA induction.

### 2.6. Co-Expression Analysis of PqMYBs and Flavonoid Biosynthesis Genes

We constructed a co-expression network of 94 flavonoid biosynthesis pathway genes and candidate *PqMYB*s in S4 and S7 subgroups based on the MeJA treatment results (Supplementary Table S9). The results revealed that *PqMYB46* exhibited the highest correlation with flavonoid pathway gene *PqANS4*, with a correlation coefficient of 0.87. Additionally, *PqMYB2* with pathway gene *PqCHS* had a correlation coefficient of 0.8, and *PqMYB46* with pathway gene *PqCCoAMT10* had a correlation coefficient of 0.82. According to the above results, we speculated that *PqMYB2*, *PqMYB46*, and *PqMYB72* may contribute to the positive regulation of flavonoid biosynthesis (Figure S4) In addition, based on the results of co-expression analysis, we selected five flavonoid pathway genes with the highest correlation with PqMYB and conducted gene expression experiments. The experimental results showed that all five genes exhibited significant responses to MeJA treatment after 24 h of treatment (Supplementary Figure S5).

### 2.7. Subcellular Localization Analysis

**Figure 7:**
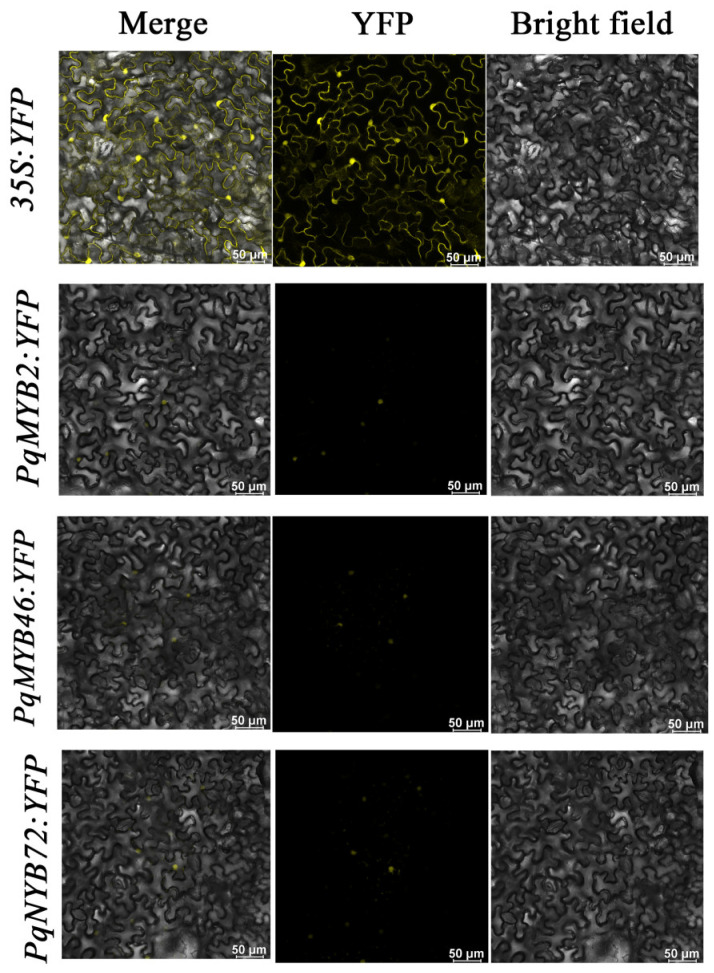
Subcellular localization of *PqMYB2*, *PqMYB46*, and *PqMYB72* in *Nicotiana benthamiana*. *35S:YFP* was used as a control. Yellow is YFP fluorescence.

We fused the candidate *PqMYBs* with the YFP reporter gene and subsequently transformed them into *N. benthamiana* leaves to investigate the localization of the three genes, *PqMYB2*, *PqMYB46*, and *PqMYB72* (Table S10). The merge represents the superimposed field; YFP is the fluorescent microscopic image excited at 514 nm, and it represents the yellow fluorescent protein and the bright-field microscopic image with cellular structures. The results indicate that compared with the distribution of the empty vector YFP in various locations within the nucleus and membrane of the cell, PqMYB2-YFP, PqMYB46-YFP, and PqMYB72-YFP only exhibited fluorescence in the cell nucleus, suggesting that PqMYB2, PqMYB46, and PqMYB72 are localized in the cell nucleus and may play regulatory roles within it (Figure 7).

## 3. Discussion

In plants, MYB transcription factors generally have one to four repeat domains, most of which contain two repeats, and because of this they belong to the R2R3-MYB family. The C-terminal is more variable and more responsible for regulation than the N-terminal. According to the conserved region of the C-terminal in *A. thaliana*, 126 R2R3-MYB proteins were divided into 26 subgroups [13]. In the biosynthesis pathways of flavonoids, the replication and subfunctionalization of the R2R3-MYB gene have promoted the functional diversity of flavonoids in angiosperms [17]. *P. quinquefolius* is an important medicinal plant within *Araliaceae*. To date, the regulation of flavonoid biosynthesis in *P. quinquefolius* is still unclear. We identified the R2R3-MYB gene family in order to explore the transcriptional regulation mechanism of flavonoid biosynthesis in *P. quinquefolius*. The physical and chemical property results from the analysis show that there are differences in amino acid sequence length, physical and chemical properties (such as molecular weight, isoelectric point, etc.), and gene structure of the *PqMYB*s in *P. quinquefolius*. This reflects the complexity and functional diversity of the *PqMYB*s. Different groups have different gene structures. Groups C24 and C27 have significantly different motif compositions from other subgroups. In contrast, Motif 6 is unique to Subgroup C24 and Motif 9 is unique to Subgroup C27, indicating that the *PqMYB*s of these two subgroups may have undergone different evolutionary development and may have specific functions (Figure 1).

Different subgroups also had different functions in the R2R3-MYB gene family. The *A. thaliana* S7 subgroup, containing *AtMYB11*, *AtMYB12*, and *AtMYB111* (AT3G62610, AT2G47460, and AT5G35550), played a regulatory role in controlling the transcription and biosynthesis of flavonoids in different parts of Arabidopsis seedlings [32]. In *Pistacia chinensis*, *PcMYB113*, which is highly similar to *AtMYB113* in sequence, has a potential role in promoting anthocyanin biosynthesis during the coloring of autumn leaves [33]. In *S. baicalensis*, SbMYB45 and SbMYB86.1 can regulate the production of flavonoids as homologous genes in the same evolutionary branch as *AtMYB12* [22]. In *A. thaliana*, *AtMYB4* in the S4 subgroup and its homologs *AtMYB7* and *AtMYB32* can also regulate the flavonoid biosynthesis pathway [34,35]. In tomato, *SiMYB7*, an analog of *AtMYB4*, was proven to inhibit the accumulation of anthocyanins in fruit [36]. *NtMYB9* was found in *Narcissus tazetta,* which belongs to the branch of *AtMYB4*, and participated in the positive regulation of flavonoid biosynthesis; it may also promote flavanol biosynthesis [37]. *FtMYB13/14/15/16*, belonging to the S4 group in *Fagopyrum tataricum*, could directly repress rutin biosynthesis. We speculate that *the PqMYB*s which are related to S4 (*PqMYB2*, *PqMYB46*, *PqMYB72*, *PqMYB84*, and *PqMYB123*) and S7 (*PqMYB5*, *PqMYB109*, *PqMYB5*, *PqMYB25*, *PqMYB51*, *PqMYB52*, and *PqMYB18*,) may also play a role in flavonoid biosynthesis. We finally selected five candidate genes of the S4 subgroup (*PqMYB2*, *PqMYB46*, *PqMYB72*, *PqMYB84*, and *PqMYB123*) for further study, according to the expression data.

The transcriptomes of *P. quinquefolius* seedlings treated with MeJA can help us to clarify the regulation mechanisms of flavonoid biosynthesis mediated by MeJA. To verify the expression of flavonoid candidate *PqMYB*s under MeJA treatment, we further verified it through a qRT-PCR experiment. The results showed that five candidate *PqMYBs* showed different degrees of response after MeJA treatment, and the changing trend of the transcription group was basically the same. The degree of response may be relevant to the accumulation of secondary metabolites, and the specific transcriptional regulatory network needs further verification.

We established a correlation analysis between the flavonoid biosynthesis pathway genes and the expression of transcription factors of *P. quinquefolius* in transcriptome sequencing, and ultimately found that most of the candidate *PqMYB*s were closely related to flavonoid biosynthesis pathway genes (the correlation was greater than 0.8). It can be seen that the candidate *PqMYB* gene has a positive regulatory effect on the pathway genes of *PqANS4*, *PqCHS*, and *PqCCoAMT10*. In *Morinda officinalis*, it was found that *MoMYB33*, which is a member of the S7 subfamily, may regulate the accumulation of flavanol. The researcher analyzed the correlation between candidate genes and pathway genes, and it was found that the expression trend of *Mo4CL2*, *MoCHI3*, and *MoFLS 4/11/12* was consistent, and the correlation was greater than 0.7 [38].

We selected three candidates *PqMYB*s with strong correlation according to MeJA hormone treatment transcriptome data to explore their subcellular location (Figure S4). In *Ginkgo biloba*, *GbMYBFL* is located in the nucleus, and it is related to the accumulation of flavonoids [39]. In *Paeonia qiui*, *PqMYBF1* is a flavanol-biosynthesis-related MYB gene and is localized to the nucleus [40]. In our study, three *PqMYBs* (*PqMYB2*, *PqMYB46*, and *PqMYB72*) with a strong correlation with flavonoid pathway genes were located in the nucleus. Our results are consistent with previous studies, in which the flavonoid-biosynthesis-related MYB was also located in the nucleus (Figure 7).

## 4. Materials and Methods

### 4.1. Plant Materials and Growth Conditions

The *P. quinquefolius* were taken from the Medicinal Garden of Jilin Agricultural University, China. We took the seedlings from the same growth stage and sprayed the leaves with 100 µm mol·mL-1 MeJA until the water drops on the surface of the leaves no longer fell. The blank control group had distilled water sprayed in addition to the same conditions. After MeJA treatment for 0, 2, 6, 12, and 24 h, we collected the leaf samples, immediately stored them at −80 °C for subsequent transcriptome sequencing and qRT-PCR, and repeated each treatment three times. *N. benthamiana* was used in the subcellular localization experiment, and it was grown in an illumination room at 22–25 °C; the light/dark period was 14 h/10 h.

### 4.2. Identification and Physicochemical Properties Analysis

Based on *P. quinquefolius* genome data [41], we used gffread (https://github.com/gpertea/gffread accessed on 16 October 2022) to extract the protein sequence from the genome and submitted it to the plantTFDB website for transcription factor identification (http://planttfdb.gao-lab.org/ accessed on 22 October 2022). Overall, 159 members of the R2R3-MYB gene were identified. The basic information about proteins, such as the theoretical isoelectric point, amino acid size, molecular weight, etc., was analyzed using the ProtParam online analysis tool in the ExPASy database (https://web.expasy.org/protparam/ accessed on 12 November 2022).

### 4.3. Phylogenetic Tree, Chromosome Location, and Collinear Analysis

The R2R3-MYB protein sequences from *A. thaliana* were downloaded from the TAIR database (http://www.arabidopsis.org/ accessed on 17 November 2022). Protein sequences of *P. quinquefolius* and *A. thaliana* R2R3-MYB were compared using Clustal W, a maximum likelihood phylogenetic tree constructed by IQTREE with 1000 bootstraps (http://www.iqtree.org/ accessed on 19 November 2022). The interspecies *P. quinquefolius* collinearity analysis of *Panax ginseng* and *Panax notoginseng* was completed using MCScanX software (version 1.1.10) and visualized with Circos software (http://circos.ca/ accessed on 26 November 2022).

### 4.4. Analysis of Motifs and Domains of Proteins and cis-Acting Elements

The conserved motifs were predicted using MEME (https://meme-suite.og/meme/tools/meme accessed on 14 September 2022) software. The conserved domains of R2 and R3 MYB in the PqMYB protein were mapped using the WEBLOGO online program (http://weblogo.berkeley.edu/logo.cgi/ accessed on 28 November 2022). Then, 2000 bp genomic DNA sequences upstream of the start codon (ATG) were submitted to the PlantCARE database to identify cis-elements in the PqMYBs promoter region (http://bioinformatics.psb.ugent.be/web-tools/plantcare/html/ accessed on 1 December 2022).

### 4.5. Expression Patterns Analysis in Different Tissues

The annotation of published *P. quinquefolius* transcriptome data CNGBdb (https://db.cngb.org/ accessed on 5 December 2022, Accession Number, CNP0001680) was carried out according to the reported *P. quinquefolius* genome [41]. We used the nf-core process to analyze and used TBtools to visualize the expression data (Supplementary Table S6) [42].

### 4.6. RNA-Seq Data and qRT-PCR

For the expression patterns of different tissues, we used RNA-seq data that we reported previously [43]. We completed the transcription sequencing for the identification of MeJA-responsive *PqMYB*s. The expression profile of R2R3-MYB is shown by the heatmap illustrator from TBtools (Supplementary Table S7). The qRT-PCR analysis method is SYBR’s two-step method, and the reaction procedure is as follows: predenaturation 94 °C, 30 s; denature 94 °C, 5 s; annealing temperature 60 °C, 30 s; 45 cycles. The reaction system is 20 μL:2 × PerfectStart^®^ Green qPCR SuperMix 10 μL, F, R primers all 1 μL, cDNA 1 μL, filled to 20 μL with ddH_2_O. The GAPDH gene was used as the internal reference gene of *P. quinquefolius* [44]. According to qRT-PCR data, the expression level of the corresponding genes was calculated using the 2^−∆∆CT^ method. The primer and reference gene sequences are shown in Supplementary Table S8.

### 4.7. Subcellular Localization Analysis

The open reading frames (ORFs) of *PqMYB2*, *PqMYB46*, and *PqMYB72* without stop codons were inserted into the *PHB-YFP* vector through homologous recombination, and the restriction sites were *HindIII* and *SacI* (Supplementary Table S10). The recombinant plasmid of kanamycin resistance fusion vector *PHB-PqMYB-YFP* with correct results after sequencing transformed into *Agrobacterium tumefaciens* strain GV 3101, and then the cultured GV 3101 strain containing the recombinant plasmid gained an OD 600 = 0.6–0.8 and was collected after centrifugation (5000 rpm, 10 min). The bacteria were suspended with the same volume of the working solution (10 mM MgCl_2_ and 100 µM acetosyringone) suspension, the OD600 was adjusted to about 0.6, and then it was injected into the lower epidermis of the tobacco leaves and the empty PHB-YFP carrier was used as the control carrier. The yellow fluorescence signal was observed with a laser confocal microscope after 48 h of incubation under low light at room temperature.

## 5. Conclusions

We identified 159 R2R3-MYBs from *P. quinquefolius* for the first time and divided them into 33 subgroups with *A. thaliana* using a phylogenetic tree. There were differences in structure and function among different subgroups. The protein statistics were 107–1050 amino acids in length, 12.21–116.44 kDa in molecular weight, and 4.57–10.34 in isoelectric point. They were distributed across 24 chromosomes, and their conserved motifs, domains, gene structures, and collinearity were analyzed with *P. ginseng* and *P. notoginseng*. In the roots, leaves, and flowers of *P. quinquefolius*, the expression patterns of 159 PqMYBs were significantly different. After MeJA treatment, the transcriptome data showed a trend of upregulation and downregulation. Five genes were selected for qPCR verification, and the experimental results were consistent with the transcriptome trend. The five candidate genes and flavonoid pathway genes have a strong correlation, indicating that candidate PqMYBs may play a certain regulatory function in the flavonoid pathway. Three genes (PqMYB2, PqMYB46, and PqMYB72) with strong correlation were selected for subcellular localization, and the results showed that they were all located in the nucleus. In subsequent scientific inquiries, the three candidate genes, namely PqMYB2, PqMYB46, and PqMYB72, will be pinpointed as crucial factors for elucidating the regulation mechanisms underlying flavonoid biosynthesis in *P. quinquefolius*. Our research provides a new theory for further analyzing the function of transcription factor R2R3-MYB of *P. quinquefolius* and provides basic data and research direction to enable a better understanding of the biosynthesis mechanism of secondary metabolites of *P. quinquefolius*.

## Data Availability

All data are contained within the article and Supplementary Materials.

## Supplementary Materials

The following supporting information can be downloaded at: https://www.mdpi.com/article/10.3390/ijms25073709/s1.
