# Supplementary material for: Identification and Expression Analysis of R2R3-MYB Transcription Factors Associated with Flavonoid Biosynthesis in Panax quinquefolius
Source: Int J Mol Sci. 2024 Mar 26;25(7):3709. doi: 10.3390/ijms25073709 (PMC11011825; doi:10.3390/ijms25073709)
Supplement: Supplementary file 1 [file ijms-25-03709-s001.zip › ijms-2909463-supplementary.pdf]

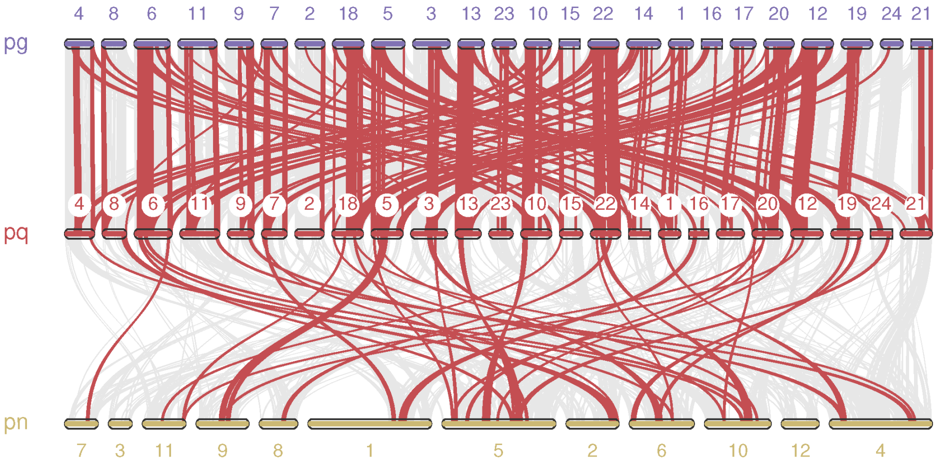

Figure S1. Synteny analyses of the R2R3-MYB genes between *P. quinquefolius* and two representative species (*P.ginseng* and *P.notoginseng)*. Gray lines represent the collinear blocks in the genomes of *P. quinquefolius* and other plant species, and red lines emphasize the syntenic PqMYBs pairs.

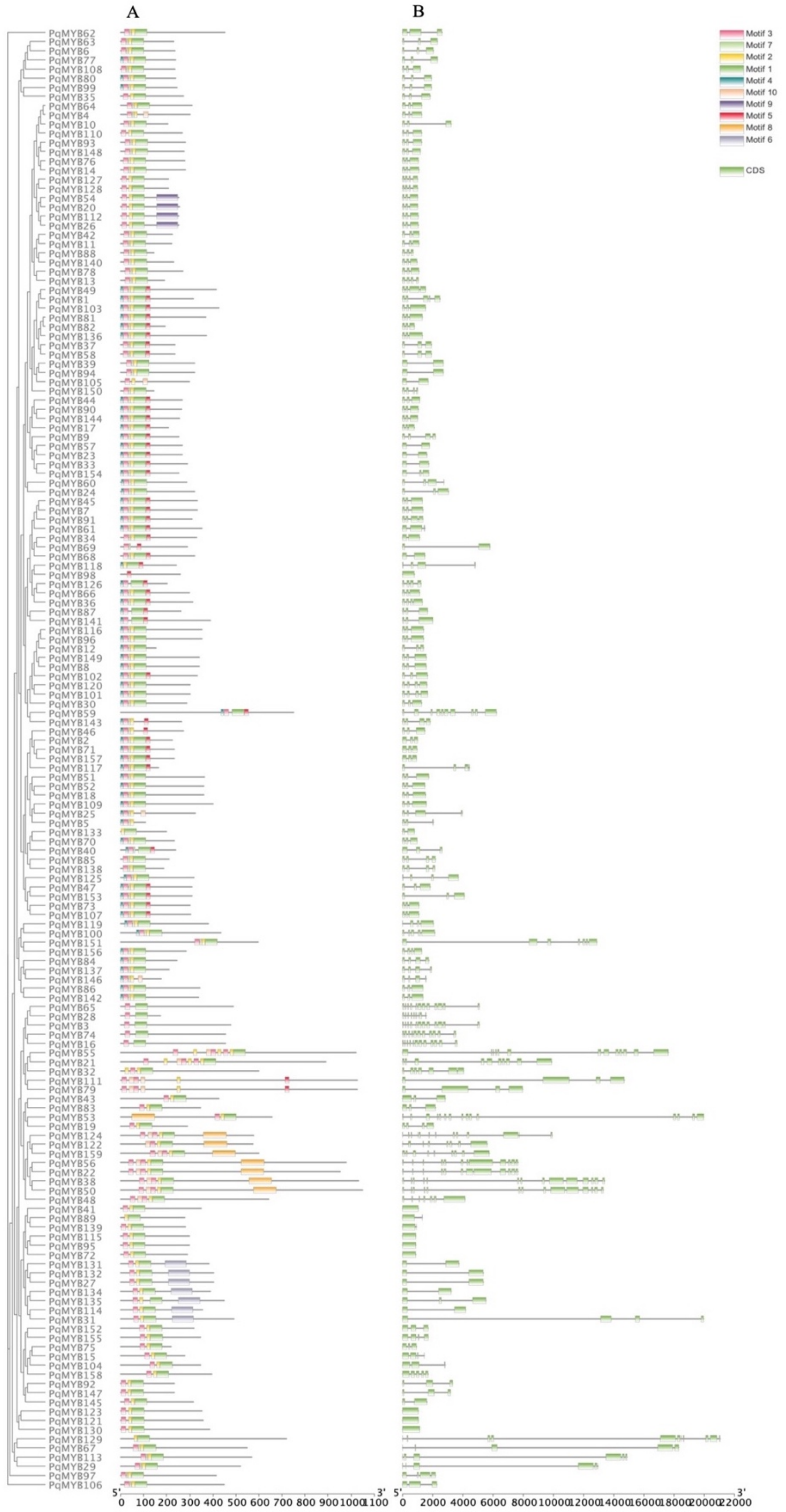

Figure S2:**A.** Analysis of conserved motif of R2R3-MYB in *P. quinquefolius* Different color blocks represent different conservative motifs **B.** The gene structure of R2R3-MYB in *P. quinquefolius*, the green box represents CDSs, and the horizontal line in the box represent introns. Motif were found using the MEME online website, and conservative domains were found using NCBI-CD-search website.

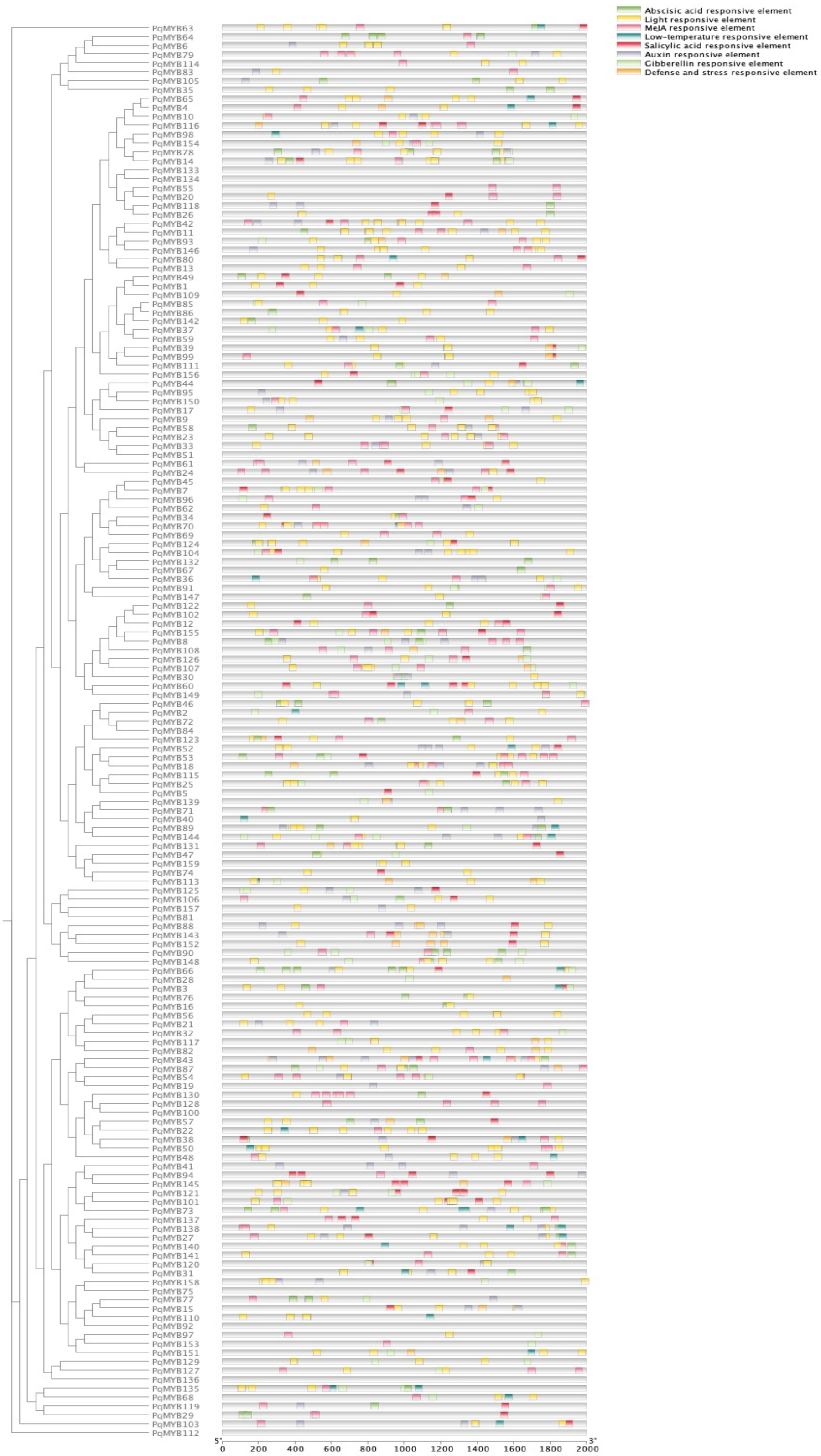

Figure S3 Cis-acting element analysis of *PqMYB* genes. Different colors on the black lines reprepresent different elements of the promoters. The online website PlantCARE was used to predict the resent different elements of the promoters.

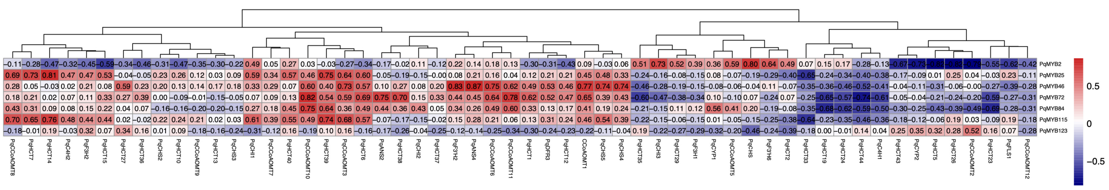

Figure S4. Correlation analysis between candidate PqMYBs and flavonoid biosynthesis pathway gene in P. q quinquefolius. Red represents a positive correlation, and the blue represents a negative correlation. The color of each color block in the figure indicates the positive or negative correlation coefficient between genes. The number in the color block indicates the absolute value of the correlation coefficient.

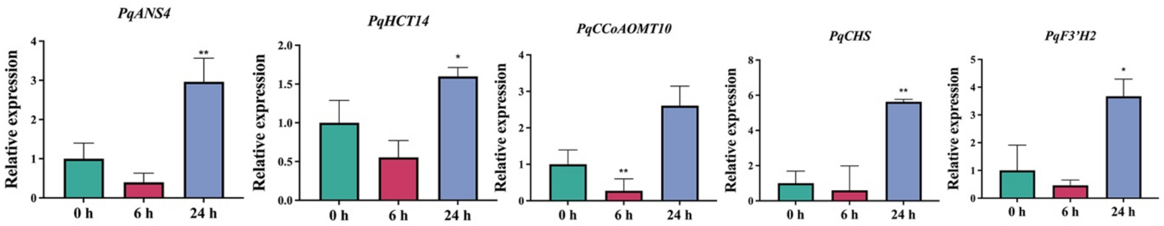

Figure S5. Relative expression of 5 flavonoid biosynthesis pathway gene in leaves of *P. quinquefolius* treated with MeJA at different times for 0 h、6 h、24 h. Data indicate the mean ± SD, and the dots represent raw data. *: $p < 0.05$. **: $p < 0.01$
